# Verletzungen des medialen Bandapparats des Kniegelenks: Wann und wie therapieren?

**DOI:** 10.1007/s00113-023-01368-z

**Published:** 2023-10-17

**Authors:** Elmar Herbst, Johannes Glasbrenner, Adrian Deichsel, Thorben Briese, Christian Peez, Michael J. Raschke, Christoph Kittl

**Affiliations:** Klinik für Unfall‑, Hand- und Wiederherstellungschirurgie, Universitätsklinikum Münster, Albert-Schweitzer-Campus 1, 48147 Münster, Deutschland

**Keywords:** Gelenkinstabilität, Innenbandruptur, Biomechanik, Konservative Behandlung, Rekonstruktive Chirurgie, Joint instability, Inner ligament rupture, Biomechanics, Conservative treatment, Reconstructive surgery

## Abstract

Verschiedene mediale Strukturen sind für die Hemmung der Valgus‑, Außenrotations- und anteromedialen Rotation zuständig. Aus Verletzungen dieser Strukturen können unterschiedlich ausgeprägte isolierte und kombinierte Instabilitäten resultieren. Das hintere Schrägband („posterior oblique ligament“, POL) wird, im Gegensatz zu früheren Spekulationen, nicht mehr als Hauptstabilisator der anteromedialen Rotationsinstabilität (AMRI) angesehen. Die akuten proximalen medialen Rupturen sind die Domäne der konservativen Therapie, mit sehr guten klinischen Ergebnissen. Im Gegensatz dazu bedingen akute distale Rupturen meistens ein operatives Vorgehen. Chronische Instabilitäten treten überwiegend in Kombination mit Instabilitäten des vorderen Kreuzbands (VKB) auf. Die klinische Untersuchung ist speziell bei diesen Instabilitäten ein wichtiger Bestandteil zur Indikationsstellung einer Operation für eine zusätzliche mediale Rekonstruktion. Bei hochgradigen medialen und anteromedialen Instabilitäten sollte an eine operative Versorgung gedacht werden. Biomechanisch erscheint eine kombinierte mediale und anteromediale Rekonstruktion den anderen Rekonstruktionsarten überlegen. Derzeit fehlen klinische Studien, um genau diesen biomechanischen Vorteil auch klinisch zu belegen.

## Abstract (en)

Different medial structures are responsible for restraining valgus rotation, external rotation, and anteromedial rotation. When injured this can result in various degrees of isolated and combined instabilities. In contrast to earlier speculation, the posterior oblique ligament (POL) is no longer considered to be the main stabilizer of anteromedial rotatory instability (AMRI). Acute proximal medial ruptures are typically managed conservatively with very good clinical results. Conversely, acute distal ruptures usually require a surgical intervention. Chronic instabilities mostly occur in combination with instabilities of the anterior cruciate ligament (ACL). The clinical examination is a particularly important component in these cases to determine the indications for surgery for an additional medial reconstruction. In cases of severe medial and anteromedial instabilities, surgical treatment should be considered. Biomechanically, a combined medial and anteromedial reconstruction appears to be superior to other reconstruction methods; however, there is currently a lack of clinical studies to confirm this biomechanical advantage.

Verletzungen des medialen Bandapparats gehören zu den häufigsten Sportverletzungen [3]. Aufgrund des ähnlichen Verletzungsmechanismus mit Valgustrauma und tibialer Rotation kommen mediale Bandverletzungen gehäuft mit Rupturen des vorderen Kreuzbands (VKB) vor [8, 21, 29, 34]. Akute, isolierte mediale Bandverletzungen können meist einer konservativen Therapie zugeführt werden [33]. Anders verhält es sich bei kombinierten Verletzungen des VKB und des medialen Bandapparats [14]. Hier herrscht in der aktuellen Literatur keine Einigkeit bezüglich der optimalen Behandlungsstrategie. Allerdings ist hinlänglich bekannt, dass eine persistierende mediale Instabilität das Risiko einer VKB-Rezidivinstabilität deutlich erhöht [2, 38].

Neben dieser rein medialen Instabilität führen Verletzungen des medialen Bandapparats allerdings auch zu kombinierten posteromedialen und v. a. anteromedialen Instabilitäten [7, 35, 40]. Diese klinisch zu detektieren, stellt eine besondere Herausforderung dar, allerdings bestimmen sie das therapeutische Vorgehen wesentlich. Basis einer operativen Therapie stellt die Kenntnis der Anatomie und elementaren Biomechanik dar, auf die im Weiteren eingegangen wird.

## Grundlagen

### Anatomie

**Figure 1:**
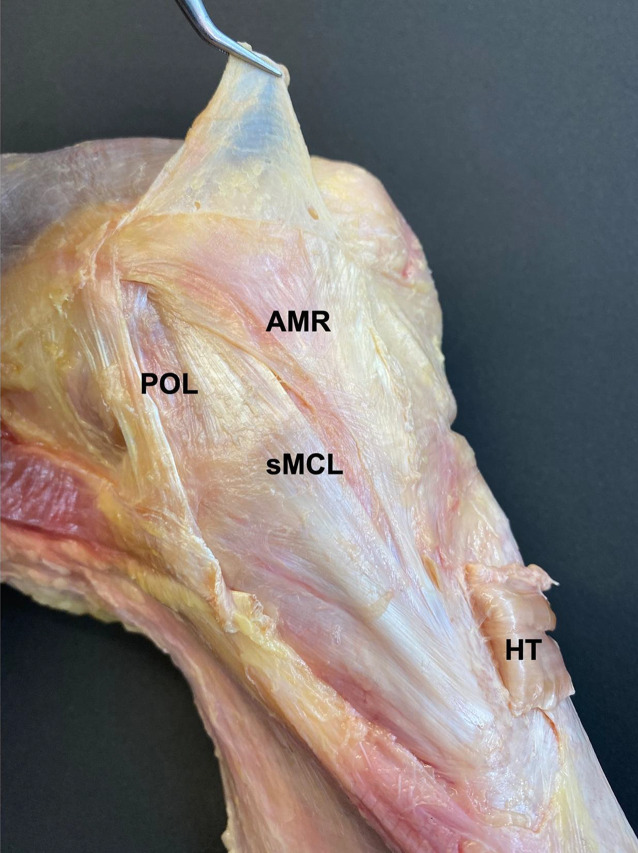

Der mediale Bandapparat besteht aus 3 bzw. 4 relevanten Strukturen: dem oberflächlichen Innenband („superficial medial collateral ligament“, sMCL), dem tiefen Innenband („deep medial collateral ligament“, dMCL), dem hinteren Schrägband („posterior oblique ligament“, POL) und im erweiterten Sinn dem anteromedialen Retinaculum (AMR) [25, 32, 36]. Im Gegensatz zur posterolateralen Gelenkecke sind die medialen ligamentären Strukturen sehr breit und flach und gehen teilweise ineinander über (Abb. 1).

Am oberflächlichsten liegt das sMCL, das am bzw. knapp distal des medialen Epicondylus entspringt und unter dem Pes anserinus superficialis hindurch nach distal anterior zur anteromedialen Tibia zieht. Auch die tibiale Insertion ist nicht punktförmig, sondern liegt flächig ca. 6–8 cm distal zum medialen Gelenkspalt [36]. Im anterioren Bereich gehen die Fasern des sMCL in das kräftige AMR über [32].

Das dMCL verläuft nicht, wie bislang beschrieben, streng von femoral nach distal, sondern fächerförmig nach anteromedial tibial und inseriert, 8 mm vom Knorpel-Knochen-Übergang entfernt und ausgehend von einem femoralen Bereich, knapp distal und posterior zum medialen Epicondylus [4].

Posteromedial erstreckt sich das POL, das knapp leicht proximal und posterior zum medialen Epicondylus entspringt und nach posteromedial verläuft. Ein Teil der Fasern inseriert an der Sehne des M. semimembranosus, wohingegen der direkte Anteil nahe der Insertion des M. semimembranosus knöchern an der Tibia inseriert [4, 25, 36].

### Biomechanik

Es ist hinlänglich bekannt, dass das sMCL der primäre Stabilisator gegen eine mediale Instabilität, also Valgusstress, ist. Dieser Funktion kommt das sMCL in allen Beugegraden, v. a. aber zwischen 30° und 90°, nach [13, 35]. Darüber hinaus wurde dem sMCL eine zusätzliche Rolle in der anteromedialen Rotationshemmung zugesprochen [40].

Das dMCL spielt eine sekundäre Rolle in der Stabilisation gegen Valgusstress [13, 35]. Allerdings wurde dem dMCL zuletzt aufgrund des fächerförmigen anatomischen Aussehens mit weit anteromedialer tibialer Insertion eine große Bedeutung in der Hemmung der tibialen Außenrotation ab ca. 30°-Flexion und der anteromedialen Rotation zugesprochen [43].

Das POL wiederum ist ein sekundärer Stabilisator gegen Valgusstress und tibialer Innenrotation in voller Extension [13, 35].

Je nach Flexionsgrad haben anteriore oder posteriore Bandanteile teils diametrale Funktionen

Die biomechanische Rolle des AMR ist noch nicht final geklärt, allerdings konnte in einer robotischen Studie gezeigt werden, dass extensionsnah die anteromediale Rotationsinstabilität (AMRI) neben dem VKB durch das AMR und die anteromediale Kapsel gehemmt wird, wohingegen erst ab 60°-Flexion das sMCL diese Rolle übernimmt [19].

Unabhängig von diesen biomechanischen Funktionen der einzelnen Bandstrukturen muss sich beim medialen Bandapparat immer vor Augen geführt werden, dass es sich um flächige Bänder handelt. Sprich, je nach Flexionsgrad haben die verschiedenen Anteile eines Bandes (anterior vs. posterior) teils diametrale Funktionen. Beispielsweise spannen sich mit zunehmender Flexion im Kniegelenk die anterioren sMCL-Fasern auf, wohingegen die posterioren Anteile erschlaffen [22]. Dies kommt insbesondere bei Rotationsinstabilitäten zum Tragen. So ist anzunehmen, dass eine AMRI, die primär in Flexion evident ist, durch Insuffizienz der weiter anterior gelegenen Kapsel-Band-Strukturen (anteromediale Kapsel, anteriore Anteile des dMCL/sMCL, AMR) hervorgerufen wird, während die posterioren Bereiche des medialen Kapsel-Band-Komplexes in diesem Kontext nur eine untergeordnete Rolle spielen [22]. Derartige Überlegungen sollten in die Planung und der chirurgischen Therapie von medialen Instabilitäten einbezogen werden, um das Transplantat entsprechend dem individuellen Instabilitätsmuster weiter nach anterior oder nach posterior zu legen.

## Diagnostik

### Anamnese und klinische Untersuchung

Neben einer fundierten Anamnese, einschließlich Unfallmechanismus und Zeitpunkt der Verletzung, ist eine fundierte klinische Untersuchung der Schlüssel zu einer möglichst patientenzentrierten Therapie. Diese beinhaltet außer der Erfassung des aktiven und passiven Bewegungsumfanges sowie des Erguss- und Schwellungszustandes die Untersuchung der ligamentären Insertionspunkte, um bereits Rückschlüsse auf die Verletzungslokalisation zu erlauben. Für die weitere Behandlung ist mitentscheidend, ob eine tibiale oder femorale mediale Bandruptur vorliegt [18].

Anschließend erfolgen Funktionstests aller Bandstrukturen am Kniegelenk (jeweils beider Kreuz- und Kollateralbänder). Für den medialen Bandapparat wird ein Valgusstresstest durchgeführt, sowohl in voller Extension, um die Integrität der posteromedialen Gelenkecke zu prüfen, als auch in 20°-Flexion, um die Stabilität des sMCL zu testen [18]. Um Rotationsinstabilitäten zu detektieren, empfiehlt sich die Durchführung einer anterioren tibialen Translation in 20°-Flexion (Lachman-Test) in tibialer Außenrotation für die AMRI. Additiv sollte eine vermehrte posteromediale Rotationsinstabilität (vermehrte hintere Schublade in tibialer Innenrotation) und AMRI (vermehrte vordere Schublade in tibialer Außenrotation) in 90°-Flexion überprüft werden [18]. Die Interpretation der Funktionstests erfolgt jeweils im Seitenvergleich.

### Bildgebende Verfahren

Die klinische Untersuchung wird durch bildgebende Verfahren ergänzt. Insbesondere bei chronischen Instabilitäten sollten Achsenstandaufnahmen durchgeführt werden, um relevante Valgusdeformitäten der unteren Extremität auszuschließen.

**Figure 2:**
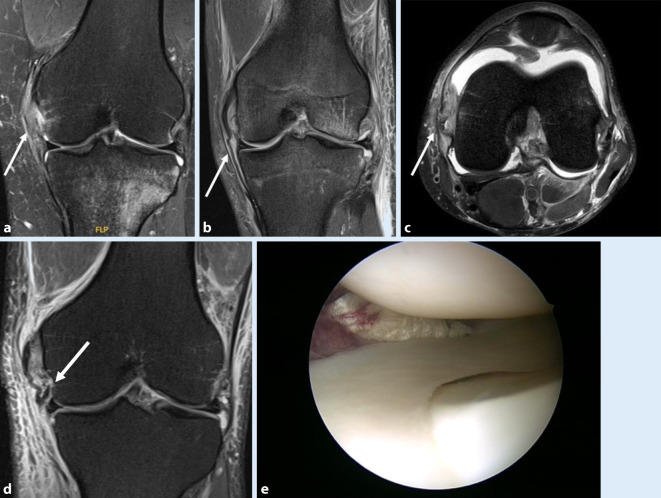

Die Magnetresonanztomographie (MRT) erlaubt neben der Darstellung von Partial- und Komplettrupturen auch die dezidierte Differenzierung zwischen femoralen und tibialen Bandrupturen [42]. Dies ist insbesondere bei tibialen sMCL-Avulsionen relevant, in deren Rahmen der Bandstumpf häufig unter den Sehnen des Pes anserinus superficialis herausgezogen wird, wodurch eine Heilung nach konservativer Therapie deutlich kompromittiert sein kann ([14]; Abb. 2). Derartige Läsionen werden als „Stener-like lesions“ bezeichnet.

In seltenen Fällen kann sich das sMCL in das Gelenk einschlagen; dies stellt eine besonders schwere Verletzung dar.

Neben den statischen bildgebenden Verfahren können auch gehaltene Aufnahmen angefertigt werden. Diese kommen vorwiegend bei den chronischen Innenbandverletzungen zum Einsatz und können einen entscheiden Hinweis auf eine Operationsindikation geben. Eine Studie an Leichenkniegelenken zeigte eine vermehrte Aufklappbarkeit des medialen Gelenkspalts von 2 mm (in voller Streckung) bis 3 mm (in 20°-Beugung) im Vergleich zum intakten Knie (Gegenseite) bei einer simulierten kompletten proximalen sMCL-Ruptur [24]. Diese Aufklappbarkeit kann sich je nach Verletzungsmuster (zusätzlich POL, VKB, hinteres Kreuzband [HKB]) erhöhen. Diese gehaltenen Aufnahmen, vorwiegend bei Multiligamentverletzungen, können auch präoperativ im OP erfolgen, um eine Refixation/Rekonstruktion zu bestätigen.

## Klassifikationen

Die traditionellen Klassifikationen teilen die medialen Instabilität in 3 bzw. 4 Grade ein und beurteilen vorwiegend die klinische Valgusaufklappbarkeit. In der Hughston-Klassifikation [20] wird die absolute Aufklappbarkeit < 5 mm (Grad I), 6–10 mm (Grad II) und > 11 mm (Grad III) beurteilt, wohingegen die Einteilung gemäß des International Knee Documentation Committee (IKDC; Grad I: 0–2 mm; Grad II 3–5 mm; Grad III: 6–10 mm; Grad IV: mehr als 10 mm) die gesunde Gegenseite als Referenz einsetzt [17]. In einer der wenigen prospektiven randomisierten Studien, die Patienten mit kombinierter VKB- und MCL-Instabilität eingeschlossen haben, wurde die Indikation zur Refixationsoperation bei der Hälfte der Patienten bei einer Instabilität über 10 mm (Grad III nach Hughston) gestellt. Des Weiteren beurteilt die IKDC die Außenrotation in 30° und 90° am Patienten in Bauchlage (Dial-Test). Hierbei wird ebenfalls auf die gesunde Seite referenziert und, gleich zur medialen Aufklappbarkeit, in 4 Grade (I: < 5°, II: 6–10°, III: 11–19°, IV: > 20°) eingeteilt. Wichtig ist jedoch, dass eine Außenrotation sowohl auf eine anteromediale Instabilität als auch auf eine posterolaterale Instabilität hindeuten kann und in der operativen Versorgung auf keinen Fall verwechselt werden sollte. Des Weiteren ist die klinische Beurteilung der Rotationsinstabilität sehr untersucherabhängig und die Differenzierung der einzelnen Grade fast unmöglich.

Eine Außenrotation kann sowohl auf anteromediale als auch auf posterolaterale Instabilität hindeuten

Müller [31] hat in seinem Buch einen neuen Klassifikationsansatz vorgestellt. Je nach verletzter Struktur wurden Instabilitätsmuster beschrieben. Hierbei war die Ruptur des POL eine Grundvoraussetzung für eine AMRI. Neuere radiologische und biomechanische Studien [5, 19, 40, 42] zeigen jedoch, dass eine POL-Ruptur selten ist und die AMRI hauptsächlich durch die medialen (sMCL, dMCL) und anteromedialen Strukturen gehemmt wird. Basierend auf diesen Daten haben Wierer et al. [40] eine biomechanische Klassifikation der AMRI erstellt. Grad 1 stellt eine reinen Rotationsinstabilität dar, die sich in den Graden 2 und 3 sukzessive erhöht. Zusätzlich besteht bei Grad 2 eine milde und bei Grad 3 eine grobe Valgusaufklappbarkeit in 30°-Kniebeugung. Diese Klassifikation ist nicht klinisch validiert und kann derzeit nicht über eine mögliche Operationsindikation Auskunft geben.

## Therapie

### Indikationen

#### Akute mediale Instabilität

Im akuten Setting der medialen Instabilität ist weiterhin die konservative Therapie der Goldstandard [11, 16, 41]. Isolierte Innenbandverletzungen werden mithilfe einer beweglichen Knieorthese und Teilbelastung behandelt; dies resultiert in zufriedenstellenden Langzeitergebnissen. Die meisten Patienten kehren nach 3 Monaten wieder zu ihrem Sport zurück [28]. Aber nicht nur die isolierte mediale Instabilität, sondern auch die Kombinationsverletzungen mit dem VKB können in der initialen Phase konservativ gut therapiert werden. Hierbei empfehlen sich, analog zur isolierten MCL-Ruptur, ein Schema mit beweglicher Orthese und Teilbelastung für 6 Wochen und erst danach das Durchführen einer potenziellen VKB-Rekonstruktion [14]. Dieses Schema erlaubt die sekundäre Evaluation einer anteromedialen/medialen Instabilität in der Narkoseuntersuchung, sodass bei nach der VKB-Rekonstruktion verbliebener medialer Instabilität eine Rekonstruktion der medialen Stabilisatoren durchgeführt werden könnte.

Auch Kombinationsverletzungen mit dem VKB sind in der initialen Phase konservativ therapierbar

**Figure 3:**
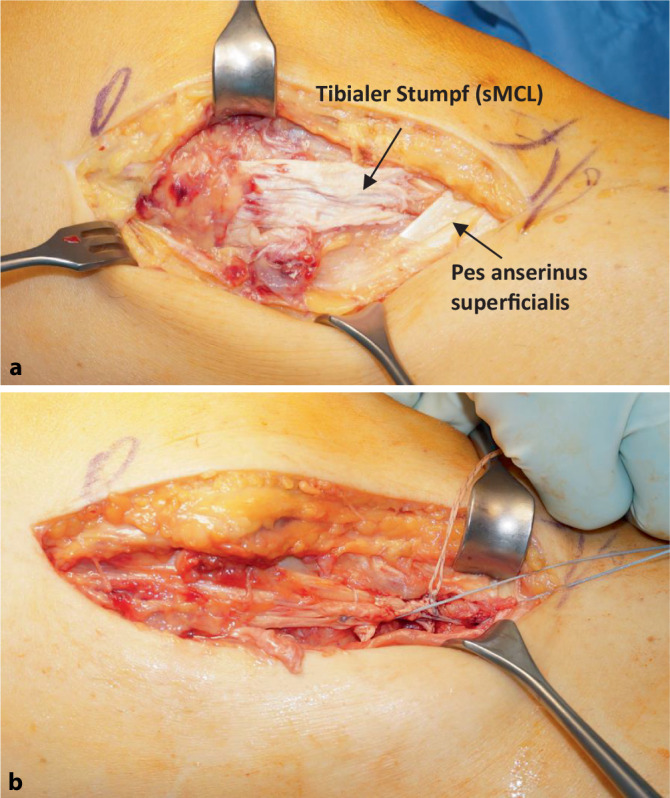

Die Indikation zur operativen Therapie der isolierten medialen Verletzung ist selten und sollte sich auf die tibialen Strip-off Verletzungen und Stener like lesions mit klinischer Aufklappbarkeit beschränken (Abb. 3). Die isolierten proximalen Rupturen sind die Domäne der konservativen Therapie, und die operative Therapie ist auf Einzelfälle beschränkt (z. B. hoher sportlicher Anspruch mit Instabilität oder offensichtliche Valgusfehlstellung). Ähnlich wie bei der isolierten Verletzung der medialen Strukturen sollte die tibiale Avulsion und die Stener-like lesion auch in Kombination mit dem VKB oder HKB operativ stabilisiert werden. Bei diesen Kombinationsverletzungen empfiehlt es sich, die Peripherie einzeitig, in seltenen Fällen früh zweizeitig (6 Wochen bis 3 Monate) mit dem zentralen Pfeiler operativ zu versorgen. Grundlage ist das „load-sharing“ der medialen Strukturen mit den Kreuzbändern [6, 37], sodass bei einer verbleibenden medialen Instabilität ein Versagen der Kreuzbandrekonstruktion befürchtet werden muss [2].

In Fällen der proximalen Rupturen der medialen Strukturen entscheidet im eigenen Vorgehen die klinische Aufklappbarkeit. Klappen die Rupturen in voller Streckung auf oder besteht eine hochgradige Instabilität in leichter Beugung, wird eine frühzeitige VKB-Rekonstruktion mit Refixation der medialen Strukturen durchgeführt. Im Gegensatz zur Valgusaufklappbarkeit ist eine Rotationsinstabilität im akuten Setting nur schwer zu beurteilen und fließt, außer in offensichtlichen Fällen, nicht in die Operationsindikationsstellung ein. In der Arthroskopie kann sich diese in einem vergrößertem Dreieck der anteromedialen Kapsel darstellen; dies bestätigt auch die frühzeitige operative Versorgung [39]. Dieses, im Vergleich zur medialen Rekonstruktion bei verbleibenden chronischen Instabilitäten, einfache Vorgehen soll einer chronischen medialen Instabilität vorbeugen. Die klinische Evidenz dazu ist allerdings sehr beschränkt. Eine prospektive randomisierte Studie zeigte bei kombinierter Versorgung des VKB (Rekonstruktion) und der medialen Strukturen (Ankerrefixation) keine signifikant unterschiedlichen Ergebnisse hinsichtlich subjektiver Scores, Beweglichkeit, Muskelkraft, „return to sports“ und VKB-Stabilität [15]. In den medialen Stressröntgenaufnahmen 2 Jahre postoperativ konnte jedoch ein signifikanter Unterschied von durchschnittlich 1,3 mm gefunden werden. Dieser Unterschied veränderte aber das postoperative Outcome der VKB-Rekonstruktion in dieser Gruppe nicht.

#### Chronische Instabilität

Da die isolierte Verletzung der medialen Strukturen die Domäne der konservativen Therapie ist und meistens keine den Patienten störende Instabilität zurückbleibt, wird in diesem Abschnitt lediglich die kombinierte VKB/MCL-Instabilität behandelt. Obwohl biomechanische Studien relativ eindeutige Hinweise auf die Krafterhöhung im VKB-Transplantat bei Valgus- oder Außenrotationsinstabilität geben [6, 37], ist die Evidenzlage in den klinischen Studien mäßig. Kohortenstudien konnten bei VKB-Rekonstruktionen mit II°-Valgus-Instabilität (IKDC-Grade B und C; 3–9 mm Valgusaufklappbarkeit im Vergleich zur Gegenseite) keine signifikanten Unterschiede bei der postoperativen klinischen Untersuchung (Lachman- und Pivot-Shift-Test), im Lysholm Score und im Return to sports zu den isolierten VKB-Rekonstruktionen finden [16, 44]. Diese Studien haben jeweils III°-Valgus-Instabilitäten (IKDC D; < 9 mm mediale Instabilität) ausgeschlossen. Basierend auf diesen Daten stellt eine chronische hochgradige (III°-)Valgus-Instabilität eine Indikation zur Rekonstruktion des VKB und der medialen Strukturen dar. Im eigenen Vorgehen werden II°-Valgus-Instabilitäten nur im Revisionsfall operativ versorgt. Dies geschieht in Anlehnung an die deutlich erhöhten Versagensraten bei präoperativer medialer Instabilität bei VKB-Revisionen [2]. Im Vergleich zu den Valgusinstabilitäten, die meistens aufgrund der verletzten Strukturen auch eine gewisse Rotationsinstabilität aufweisen, sind reine Rotationsinstabilitäten noch weniger in der Literatur beleuchtet. Derzeit gibt es keine klinische Evidenz für eine operative Therapie bei isolierten AMRI.

### Konservative Maßnahmen

Die konservative Therapie beinhaltet die initiale Ruhigstellung in der immobilisierenden Knieorthese für ungefähr eine Woche. Diese kann auch in einer 10°-Beugestellung eingestellt werden. Danach kommt eine bewegliche Knieorthese zum Einsatz. Im eigenen Vorgehen wird diese auf 0‑0-60°, 0‑0-90° und 0‑0-frei für jeweils 2 Wochen limitiert. Die passive Beweglichkeit (Physiotherapie, „continuous passive motion“) kann nach einigen Tagen schmerzadaptiert mit einer Valgusprotektion erfolgen. Es schließt sich die 20-kg-Teilbelastung für 4 Wochen an [10].

In Kniegelenken mit einem Valgus-Alignment kann dieses Regime angepasst werden. Hierbei kann die bewegliche Ortheseneinstellung auf 0‑20-60° und 0‑10-90° modifiziert werden. Außerdem kann für die ersten 4 Wochen eine vollständige Entlastung erfolgen. Dieses Regime wird sowohl für die isolierten als auch für die kombinierten (operativ und konservativ) Instabilitäten angewandt.

### Operative Versorgung

#### Refixation

Für die Refixation im akuten Setting stehen moderne Ankersysteme zur Verfügung. Im eigenen Vorgehen wird bei proximalen Rupturen ein „All-suture“-Anker und bei den distalen Rupturen ein Schraubanker verwendet. Die Insertionsanatomie sollte, speziell bei proximalen Rupturen, respektiert werden, da nur kleine Abweichungen zu Längenveränderungen [23] und wahrscheinlich dadurch auch zu veränderten Spannungsverhältnissen führen können. Bei den distalen Rupturen kann der Stumpf teilweise nicht mehr an der anatomischen Insertionsstelle unter dem Pes anserinus superficialis refixiert werden. Basierend auf Längenveränderungsstudien erscheint eine Refixation weiter proximal nicht problematisch [12].

Längenveränderungen sollten mithilfe der Isometriemessung verifiziert werden

Eine zusätzliche Augmentation mithilfe einer Sehne oder eines hochreißfesten Fadens kann bei schlechtem Bandgewebe überlegt werden. Hierbei sollte jedoch, wie bei einer Rekonstruktion, die Längenveränderungen mithilfe der Isometriemessung verifiziert werden. Eine Verlängerung des Konstrukts in Beugung sollte vermieden werden, da es zu einer Beugehemmung und Arthrofibrose, mit katastrophalem Outcome für den Patienten kommen kann. Besonders wichtig ist dies beim „internal brace“, weil das Fadenkonstrukt, aufgrund der erhöhten Steifigkeit, mit einem „overconstrainment“ und einer gestörten Kniegelenkkinematik einhergehen kann. Intraligamentäre Rupturen sollten genäht und das Nachbehandlungsschema auf eine konservativere Variante angepasst werden [14]. Außerdem sollte beachtet werden, dass eine Kombinationsverletzung von sMCL, dMCL und POL vorliegen kann. Die Lokalisation der dMCL-Ruptur kann mithilfe der Arthroskopie verifiziert werden. Hierbei hebt sich der mediale Meniskus je nach Ruptur vom Tibiaplateau (distale Ruptur) oder vom Femur (proximale Ruptur) ab.

Potenzielle Komplikationen bei den Refixationen beinhalten postoperative Schmerzen, Bewegungseinschränkungen mit konsekutiver Arthrofibrose und „overconstrainment“ bei „Internal-brace“-Augmentation (Tab. 1).

**Table 1:** 

Komplikation/„pitfall“	Potenzielle Lösung
Tunnelkonflikt bei gleichzeitiger HKB-Rekonstruktion	Femorale Bohrung im 30°-Winkel nach proximal und anterior neigen
Femorale Fehlinsertion (auch Refixation)	Intraoperative Isometriemessung oder Bestimmung des Insertionspunktes im streng seitlichen Röntgenbild
Abscheren des Transplantats bei Interferenzschraubenfixation	Bohren des Tunnels, speziell tibial, in leichter Angulation nach distal
Bewegungseinschränkung und Arthrofibrose	Anbindung an Physiotherapie, regelmäßige Nachkontrollen, orales Kortisonschema

*HKB* hinteres Kreuzband

#### Rekonstruktion

In einem systematischen Review evaluierte Delong et al. [9] das klinische Outcome der damals verfügbaren medialen Rekonstruktionen und fand eine Überlegenheit der anatomischen Rekonstruktionen (sMCL + POL) im Vergleich zu den nichtanatomischen Verfahren. Laprade et al. [26] z. B. konnten mit ihrer Rekonstruktion bei 28 Patienten die Valgusinstabilität von 6,2 mm auf 1,3 mm 6 Monate postoperativ reduzieren. Ähnliche Ergebnisse konnte auch Lind [27] mit seiner femoralen Eintunnelrekonstruktion erzielen. Es wiesen 98 % der 61 Patienten einen normalen oder fast normalen IKDC Score auf. In einer retrospektiven Analyse von fast 500 Patienten, bei denen eine kombinierte VKB/mediale Instabilität vorlag, zeigten Lind et al. [27] jedoch, dass die Valgusstabilität nur in 69 % der Fälle komplett wiederhergestellt werden konnte. Diese Ergebnisse lassen auf das Verbesserungspotenzial bei den medialen Rekonstruktionen schließen.

Derzeitige Rekonstruktionen zielen auf die Hemmung der Valgus- und der anteromedialen Instabilität ab

**Figure 4:**
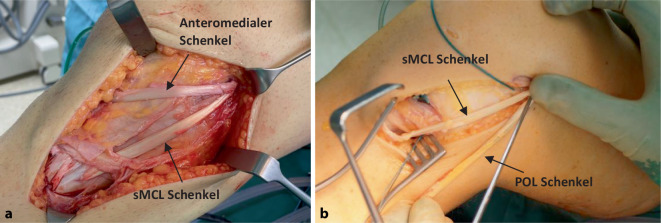

Die Erkenntnisse befinden sich derzeit, aufgrund neuer biomechanischer Erkenntnisse, die das POL nicht mehr als Hauptstabilisator der AMRI und medialen Instabilität beschreiben, im Wandel. Dieser Wandel führt dazu, dass die derzeitigen Rekonstruktionen versuchen, neben der Valgusinstabilität auch eine anteromediale Instabilität zu hemmen ([1, 30, 39]; Abb. 4). Dies geschieht mithilfe eines anteromedialen Schenkels, der das dMCL/anteromediale Retinaculum rekonstruiert und eine AMRI bei kombinierter VKB/medialer Instabilität hemmt. Der theoretische Vorteil einer zusätzlichen anteromedialen Stabilisierung kann derzeit nur in biomechanischen Studien bestätigt werden [7].

Im eigenen Vorgehen wird bei den anteromedialen Kombinationsverletzungen eine femorale Eintunnelrekonstruktion mit sMCL und anteromedialem Schenkel durchgeführt. Als Transplantat kann die Semitendinosus‑, Grazilissehne, ein „peroneus split graft“ oder ein Allograft verwendet werden. Eine Isometriemessung oder das Aufsuchen des femoralen Insertionspunkts mithilfe des streng seitlichen Röntgens ist essenziell. Wie schon bei den Refixationen beschrieben, können nur kleine Abweichungen von der femoralen anatomischen Insertion zu einem Overconstrainment in Beugung führen [23]. Im eigenen Vorgehen wird eine femorale Insertion leicht posterior des medialen Epicondylus gewählt, um eine Valgusstabilisierung des sMCL in Streckung zu erreichen. Der anteriore Schenkel wird ungefähr 2 cm distal des Gelenkspalts und 1 cm posterior der Tuberositas tibiae platziert. Auf einen Konflikt mit dem tibialen VKB-Tunnel ist zu achten. Zur Fixierung stehen moderne Anker-, „Adjustible-loop-button“- und Interferenzschraubensysteme zur Verfügung. Je nach Platzierung sollte auch die Anspannung erfolgen. Im oben genannten Vorgehen wird der sMCL- und anteromediale Schenkel jeweils in 30°-Beugung angespannt. Eine mögliche POL-Rekonstruktion, um bei kombinierten HKB-Instabilitäten eine vermehrte posteromediale Instabilität zu hemmen, sollte in voller Streckung angespannt werden, um eine Streckhemmung zu vermeiden.

Potenzielle Komplikationen bei den Rekonstruktionen sind Tunnelkonflikte, falsche femorale Insertionsstellen und Abscheren des Transplantats (Tab. 1).

## Ausblick

**Figure 5:**
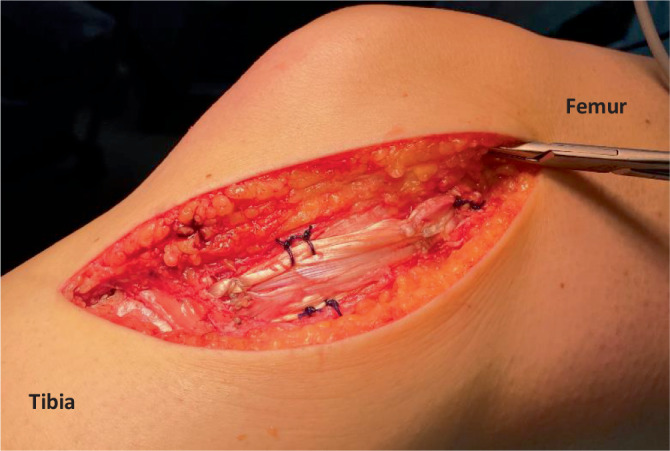

Biomechanische Studien demonstrieren, dass die einzelnen Fasern des sMCL verschiedene Arten von Instabilitäten hemmen [19]. Es konnte z. B. gezeigt werden, dass die anterioren Fasern einen wesentlichen Anteil an der Hemmung der AMRI und Außenrotation haben. Diese verschiedenen Instabilitäten können aber nicht durch eine „Single-bundle“-sMCL-Rekonstruktion gehemmt werden, sodass nur eine flache Rekonstruktion dieses Verhalten des nativen sMCL nachahmen könnte (Abb. 5) [7]. Folglich bestehen erste Bestrebungen, die runden Sehnen aufzusplitten und eine flache, „anatomische“ Rekonstruktion zu entwickeln. Ein weiterer Vorteil, speziell für klinische Studien, ist die Quantifizierung einer AMRI. Diese kann mit den derzeitigen Untersuchungsmethoden nicht suffizient evaluiert werden. Die Entwicklung einer standardisierten Methodik, um eine AMRI zu analysieren und klassifizieren, könnte das Potenzial der neueren Rekonstruktionsmethoden besser analysieren.

## Fazit für die Praxis

- Verschiedene mediale Strukturen sind für die Hemmung der Valgus‑, Außenrotations- und anteromedialen Rotation zuständig. Aus ihren Verletzungen können unterschiedlich ausgeprägte isolierte und kombinierte Instabilitäten resultieren.
- Akute, isolierte mediale Bandverletzungen können meist konservativ therapiert werden.
- Neben der rein medialen Instabilität können Verletzungen des medialen Bandapparats auch zu kombinierten posteromedialen und v. a. anteromedialen Instabilitäten führen. Diese sind klinisch schwer zu detektieren.
- Die klinische Untersuchung wird daher durch bildgebende Verfahren, wie MRT und gehaltene Aufnahmen, ergänzt.
- Aufgrund neuer biomechanischer Erkenntnisse, die das hintere Schrägband (POL) nicht mehr als Hauptstabilisator der anteromedialen Rotations- und medialen Instabilität identifizieren, wird im Rahmen derzeitiger Rekonstruktionen versucht, neben der Valgusinstabilität auch eine anteromediale Instabilität zu hemmen.
